# Higher prevalence of pulmonary macrothrombi in SARS‐CoV‐2 than in influenza A: autopsy results from ‘Spanish flu’ 1918/1919 in Switzerland to Coronavirus disease 2019

**DOI:** 10.1002/cjp2.189

**Published:** 2020-11-13

**Authors:** Nina Maria Burkhard‐Koren, Martina Haberecker, Umberto Maccio, Frank Ruschitzka, Reto A Schuepbach, Annelies S Zinkernagel, Thomas Hardmeier, Zsuzsanna Varga, Holger Moch

**Affiliations:** ^1^ Department of Pathology and Molecular Pathology University Hospital Zurich Zurich Switzerland; ^2^ Department of Cardiology University Heart Center, University Hospital Zurich Zurich Switzerland; ^3^ Institute for Intensive Care Medicine University Hospital Zurich Zurich Switzerland; ^4^ Department of Infectious Diseases University Hospital Zurich Zurich Switzerland

**Keywords:** influenza A, COVID‐19, pulmonary embolism, autopsy

## Abstract

Similar to the influenza A pandemic in 1918/1919, the new Coronavirus disease 2019 (COVID‐19) has spread globally. The causes of death in COVID‐19 are frequently compared to a seasonal influenza outbreak. Complete COVID‐19 autopsy studies were almost non‐existent in the first months of the outbreak and are still rare with respect to the number of deaths. It has been recently reported that capillary microthrombi are significantly more prevalent in patients with COVID‐19 than in patients with influenza A. To date, the contribution of macrothrombi, i.e. visible thrombi in pulmonary arteries, to the death of patients with influenza A in comparison to COVID‐19 remains unaddressed. Here, we report autopsy findings in 411 patients who died from the ‘Spanish’ influenza A pandemic between May 1918 and April 1919 at the University Hospital Zurich, Switzerland. We compare these results with influenza A autopsies from 2009 to 2020, other influenza A autopsy series and all COVID‐19 autopsies published to date. No descriptions of any macroscopic thromboembolic events were mentioned in influenza A autopsy reports. In 75 published COVID‐19 autopsies, pulmonary artery thrombosis/embolism was reported in 36%. The direct comparison of macroscopic autopsy findings suggests a significantly greater degree of grossly visible pulmonary macrothrombi in patients with COVID‐19 in comparison to influenza A autopsies even though most patients received empiric thromboprophylaxis. This is consistent with the concept of a SARS‐related *de novo* coagulopathy with generalised *in situ* clot formation, which could explain the high incidence of pulmonary thrombosis/embolism with or without underlying deep vein thrombosis and in the absence of a history of venous thromboembolic events.

## Introduction

The influenza A pandemic in 1918/1919 killed approximately 50 million people worldwide. This global outbreak was caused by a new strain of the influenza A virus. The new Coronavirus disease 2019 (COVID‐19) has also spread globally with more than 440 000 reported deaths. The causes of death in COVID‐19 are frequently compared with a seasonal influenza outbreak, although complete COVID‐19 autopsy studies were almost non‐existent in the first months of the outbreak and are still rare relative to the number of deaths. In COVID‐19 patients, a progressive life‐threatening pneumonia and a high incidence of venous thromboembolic events (VTE) have been observed [1, 2, 3]. Patients with influenza A (H1N1) infection can also die from a pneumonia, but the new coronavirus SARS‐Cov‐2 results in a more severe disease course as compared to the 2009 seasonal flu [4]. Recently, Ackermann *et al* [5] reported a distinct pulmonary pathobiology observed in Covid‐19 patients when compared to equally severe H1N1 virus infections. Capillary microthrombi were significantly more prevalent in patients with COVID‐19 than in patients with influenza A. However, the contribution of macrothrombi, i.e. visible thrombi in pulmonary arteries, to the death of patients with influenza A in comparison to COVID‐19 remains unaddressed to date.

## Methods

We summarise autopsy findings in 411 patients who died from the ‘Spanish’ influenza A pandemic 1918/1919 at the University Hospital Zurich, Switzerland [6]. In these years, the University Hospital Zurich hospital reported an influenza A lethality of 13% and autopsies were performed in almost all deceased patients. All autopsies in Zurich were performed by experienced pathologists (e.g. Prof. von Meyenburg). We reviewed all pathological data in the autopsy reports for macroscopic findings in the heart, lungs, intestines, brain, kidneys and liver. These findings were compared to the autopsy results from patients with influenza A infection in the Department of Pathology, Zurich between 2009 and 2020. In addition, we summarised all macroscopic findings from COVID‐19 autopsies published up until July 2020.

## Results

**Figure 1:**
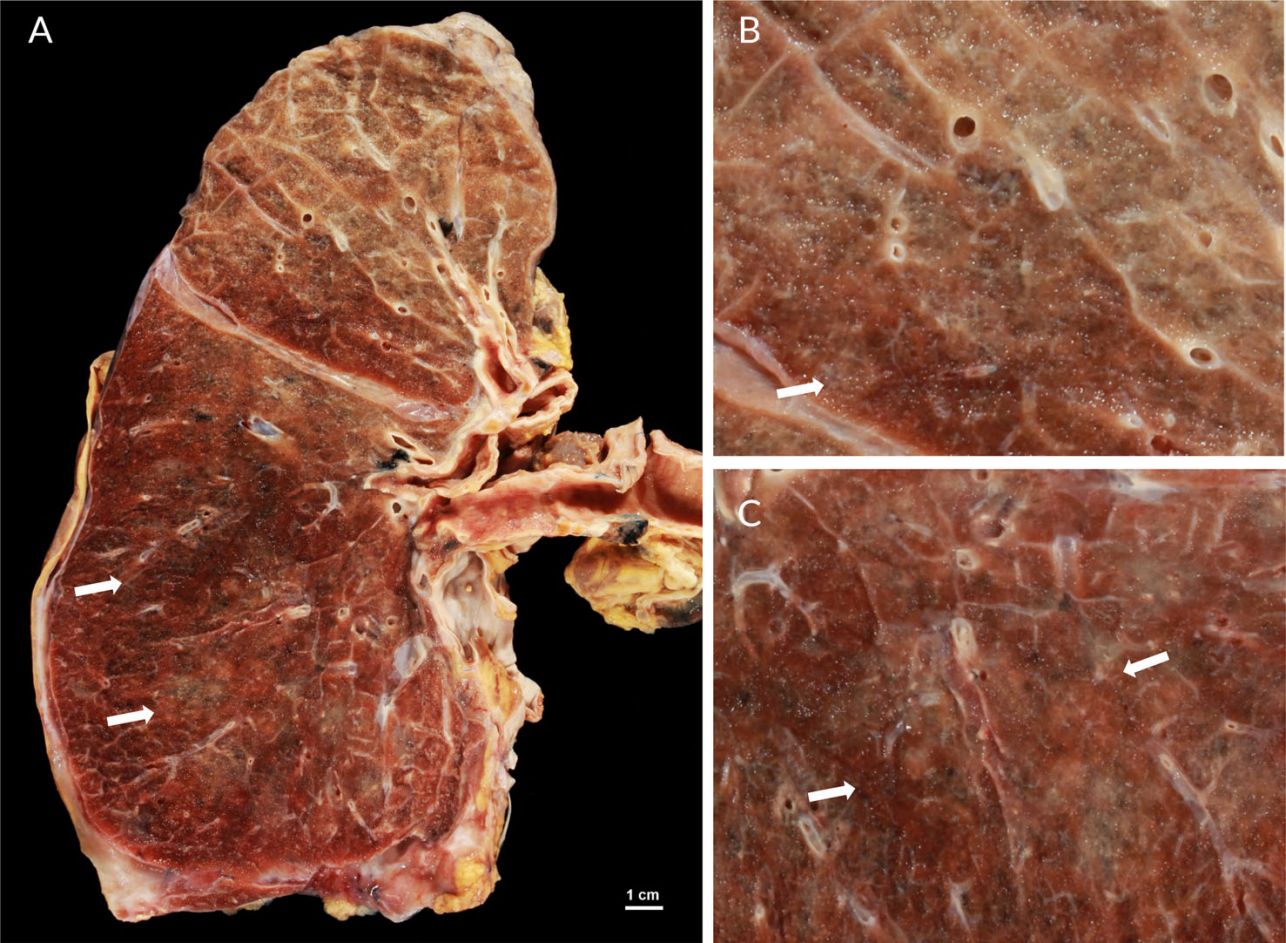
Post‐mortem pulmonary gross findings in an influenza A patient. (A) Gross appearance of the lung of a 61‐year old female patient showing diffuse haemorrhagic consolidation suggestive of bronchopneumonia, predominantly in the lower lobe (arrows). (B,C) Higher magnification from the same lung reveals open vessels and consolidation (arrows).

**Figure 2:**
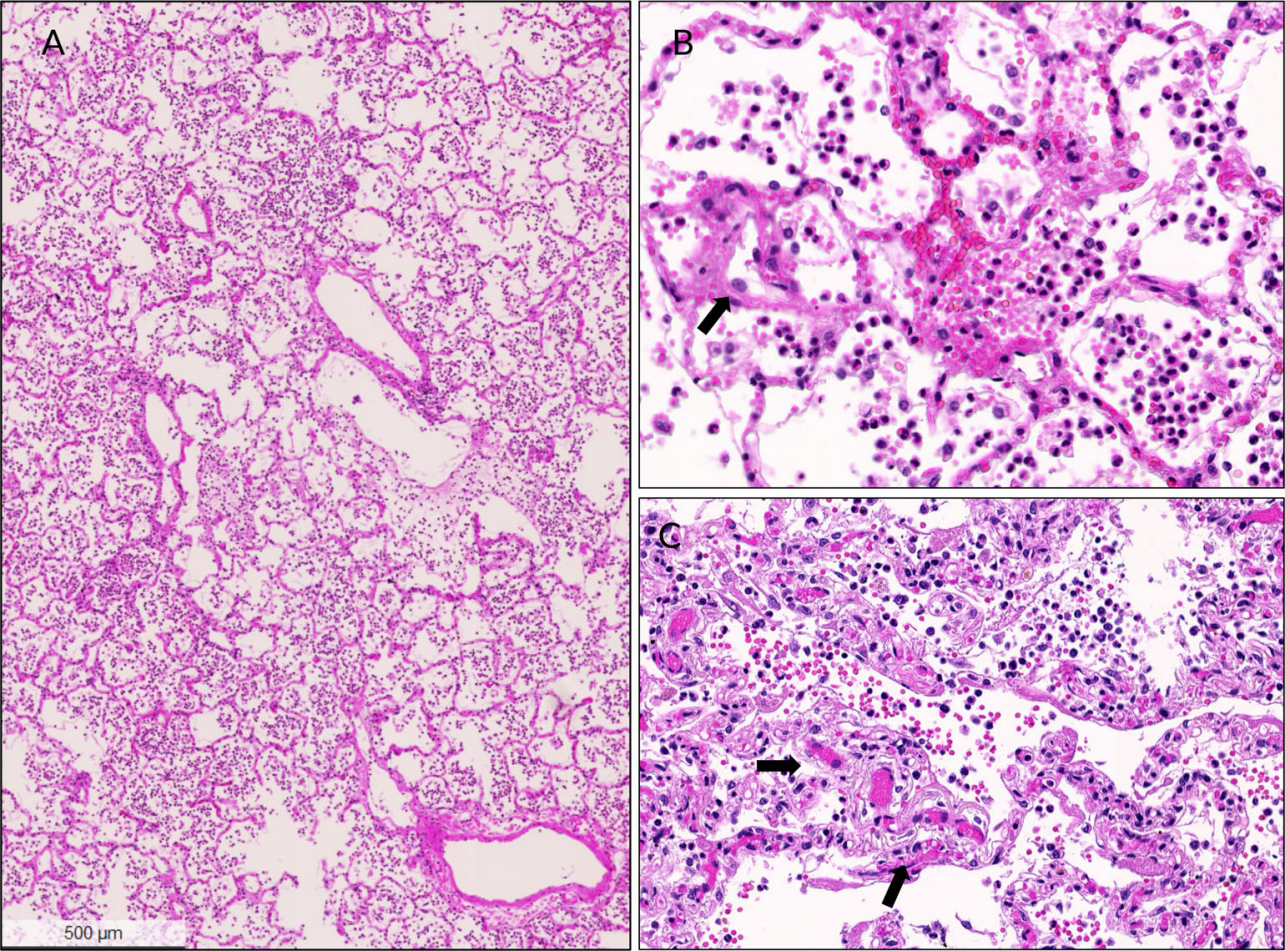
Histopathological findings in lungs of influenza A patients. (A) Lung of a 22‐year old female with influenza A infection. There are acutely congested alveolar spaces and interstitial capillaries, and many granulocytes related to secondary bacterial pneumonia in the alveolar spaces in a patchy distribution pattern. (B) High power view of the same patient shows congested vessels, intra‐alveolar bronchopneumonia and scattered reactive enlarged endothelial cells (arrow) without any evidence of endotheliitis. (C) Higher power view of a lung from a 84‐year old female patient with influenza A showing congested vessels (arrows) and intra‐alveolar bronchopneumonia. There is no evidence of fibrin thrombus formation. All images H&E stains.

In 411 of 970 autopsies between May 1918 and April 1919, influenza was noted as the cause of death in the official death certificate, with most cases arising in July 1918 and a second wave occurring between October and December 1918. In Zurich, the mortality rate was significantly higher in the second wave compared to the first wave. The age distribution showed a W‐shaped age‐curve, which exhibited peaks in infancy, between 20 and 30 years of age and in elderly individuals (range, 1–85 years), dissimilar to the SARS‐CoV‐2 pandemic affecting older patients. 62% of patients were male. Most of the patients were of normal weight or underweight (Table 1). For all age groups, death was always associated with pneumonia and related pulmonary complications (Table 2). Other important findings include a high prevalence of inflammation in the upper respiratory tract (tracheitis, bronchitis), as well as pleural effusion and pleuritis. Splenomegaly was noted in 37%. Heart hypertrophy occurred in only 16.3% of all autopsies. At that time, hypertrophy was defined as a larger size compared to a person's fist, or an increased weight. The average weight of a human heart was 300 g in the male and 250 g in a female. From 2009 to 2020, we performed 12 autopsies on patients with influenza A infection (mean age 46 years; range 1–84 years). Eleven of 12 patients had bronchopneumonia (Figures 1 and 2). We did not observe macroscopic pulmonary artery thrombosis (Table 1).

**Table 1:** Autopsy findings in 411 influenza A patients from 1918/1919, 12 influenza A patients from 2009 to 2020 and 75 published COVID‐19 patients.

	Influenza A (1918/1919)	Influenza A (2009–2020)	COVID‐19 (2019/2020)
Mean age (range), years	28 (1–85)	46 (1–84)	70 (34–96)
Male patients (%)	255 of 411 (62)	3 of 12 (25)	51 of 71 (72)
Obesity (%)*	53 of 333^‡^ (16)	0	25 of 60^‡^ (42)
Underweight (%)^†^	107 of 333^‡^ (32)	0	1 of 60^‡^ (2)
Bronchopneumonia (%)	411 (100)	11 of 12 (92)	25 of 75 (33)
Pulmonary arterial thrombosis/embolism (%)	0	0	27 of 75 (36)
Pulmonary infarction (%)	0	0	14 of 75 (19)
DVT (%)	NA	NA	9 of 25^‡^ (36)
Acute myocardial infarction (%)	0	0	2 of 71^‡^ (3)
Mesenteric infarction (%)	0	0	3 of 39^‡^ (8)

^*^ Obesity was defined as BMI >30 for influenza A patients 2009–2020 and COVID‐19 patients 2019/2020; obesity was defined as thickness of abdominal fat layer >3 cm for 1918/1919 influenza patients >20 years (no data for 19% of patients).

^†^ Underweight was defined as <18.5 kg/m^2^ for COVID‐19 patients; underweight was defined as thickness of abdominal fat layer <1 cm for 1918/1919 influenza patients >20 years (no nutritional data for 19% of patients).

^‡^ Absolute numbers can differ due to non‐available information in original publications. DVT, deep vein thrombosis; NA, not available.

**Table 2:** Autopsy pathology of 411 influenza patients, 1918/1919 (only macroscopic findings).

	%
*Upper respiratory tract*	
Laryngitis	20.7
Laryngeal ulcers	0.5
Tracheitis	78.3
Bronchitis	78.8
*Lower respiratory tract*	
Pneumonia	100
Pleuritis	66
Pleural effusion	51
Pleural empyema	12
Pneumothorax	2
Emphysema	15
*Heart*	
Endocarditis	3.4
Myocarditis	0.5
Pericarditis	5.1
Aortic valve disease	6.1
Mitral valve disease	5.6
Ventricular dilation	21.2
Cardiac hypertrophy	16.3
Atrial septal defect	0.2
*Gastrointestinal tract*	
Pharyngitis	18
Oesophagitis	1
Gastritis	15
Gastric/duodenal ulcers	3
Peritonitis	3
Colitis	3
*Liver*	
Liver necrosis	15
Cirrhosis	1
Icterus	7
Steatosis	9
*Brain*	
Encephalitis	0
Meningitis	2.4
Abscess	0.2
Haemorrhage	0.5
Cystic infarct	0.2
*Lymphatic organs*	
Lymph node hyperplasia	15
Tonsillar hyperplasia	21
Splenomegaly	37

19% of female patients were pregnant or post‐partal. No specific renal findings were reported. The presence of tuberculosis was noted in 8% of the patients.

**Figure 3:**
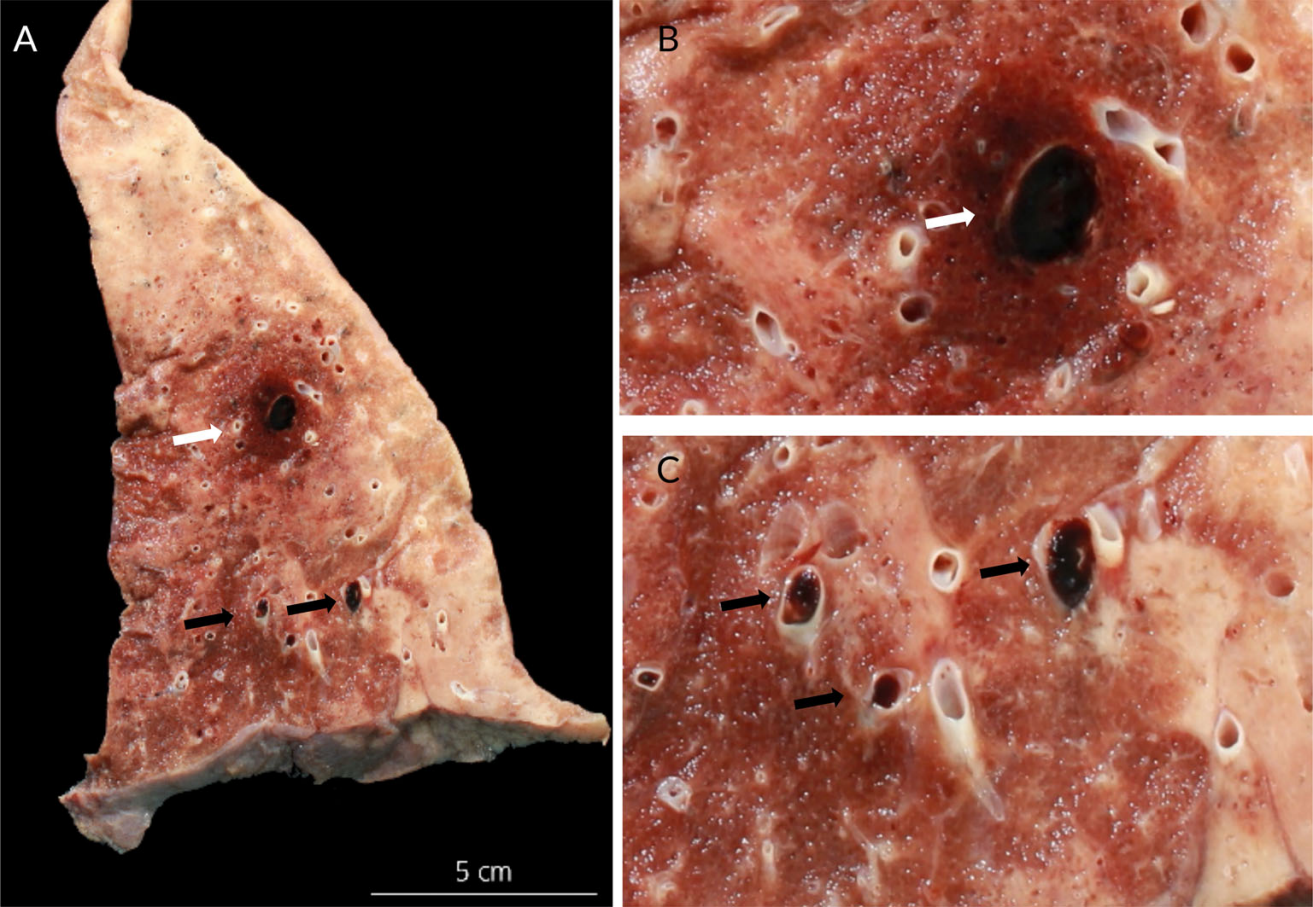
Post‐mortem pulmonary gross findings in a COVID‐19 patient. (A) Lung of a 81‐year old male patient with coronary heart disease and arterial hypertension showing several thrombotic occlusions of large (white arrow) and medium sized (black arrows) arteries. Higher power views of thrombotic occlusion of (B) a large pulmonary artery (white arrow) and (C) medium sized arteries (black arrows).

**Figure 4:**
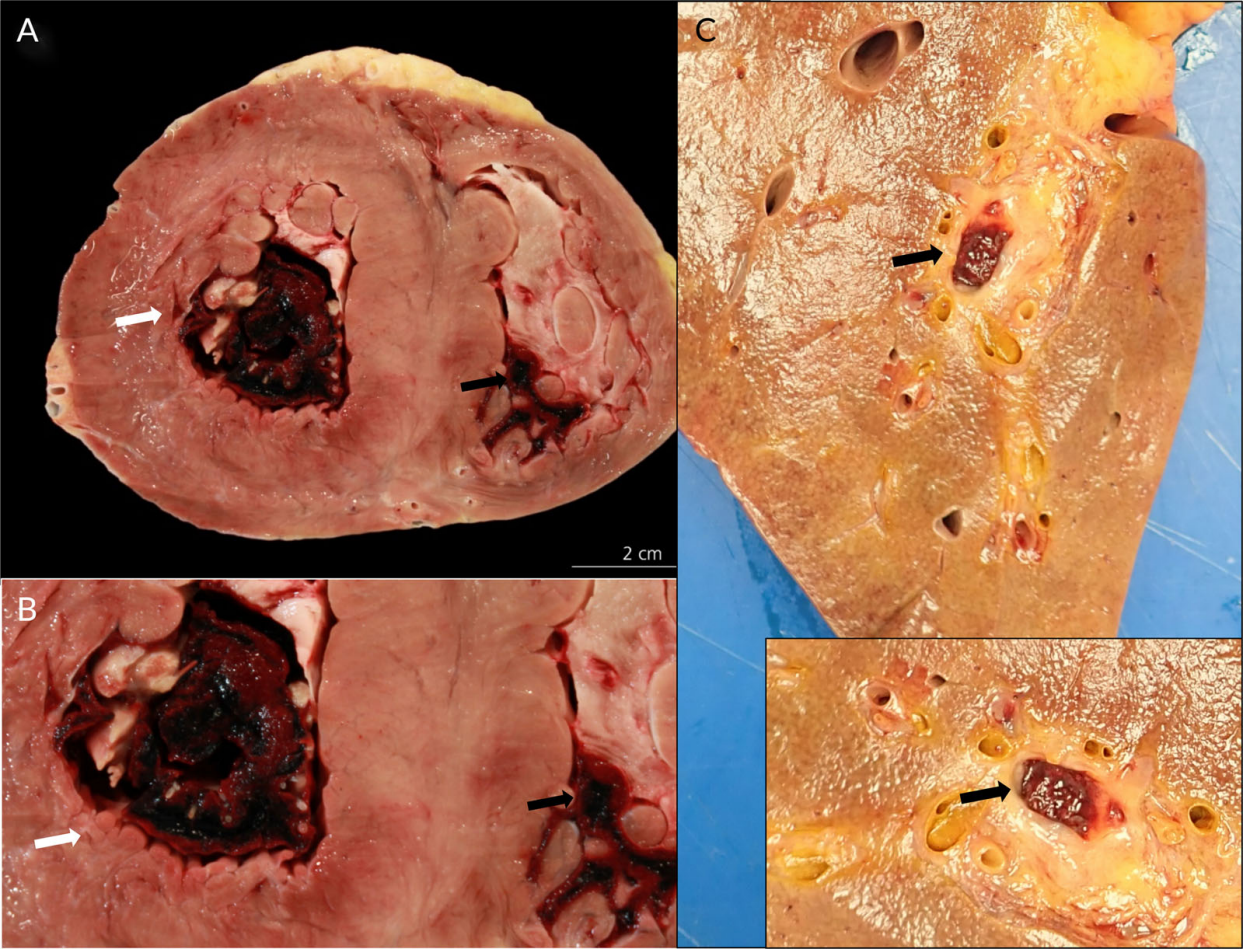
Post‐mortem cardiac and hepatic gross findings in COVID‐19 patients. (A) Cardiac macrothrombosis in a 54‐year old male patient with a history of renal transplantation due to diabetic nephropathy who developed progressive respiratory failure despite mechanical ventilation. There are large thrombi in the left (white arrow) and right (black arrow) ventricles. (B) Higher power view of intra‐ventricular thrombus in the left (white arrow) and right (black arrow: proportion of histologically confirmed thrombus) ventricles. (C) Liver of a 76‐year old male patient showing a large venous thrombus (arrow). Inset: Higher power view (rotated).

**Figure 5:**
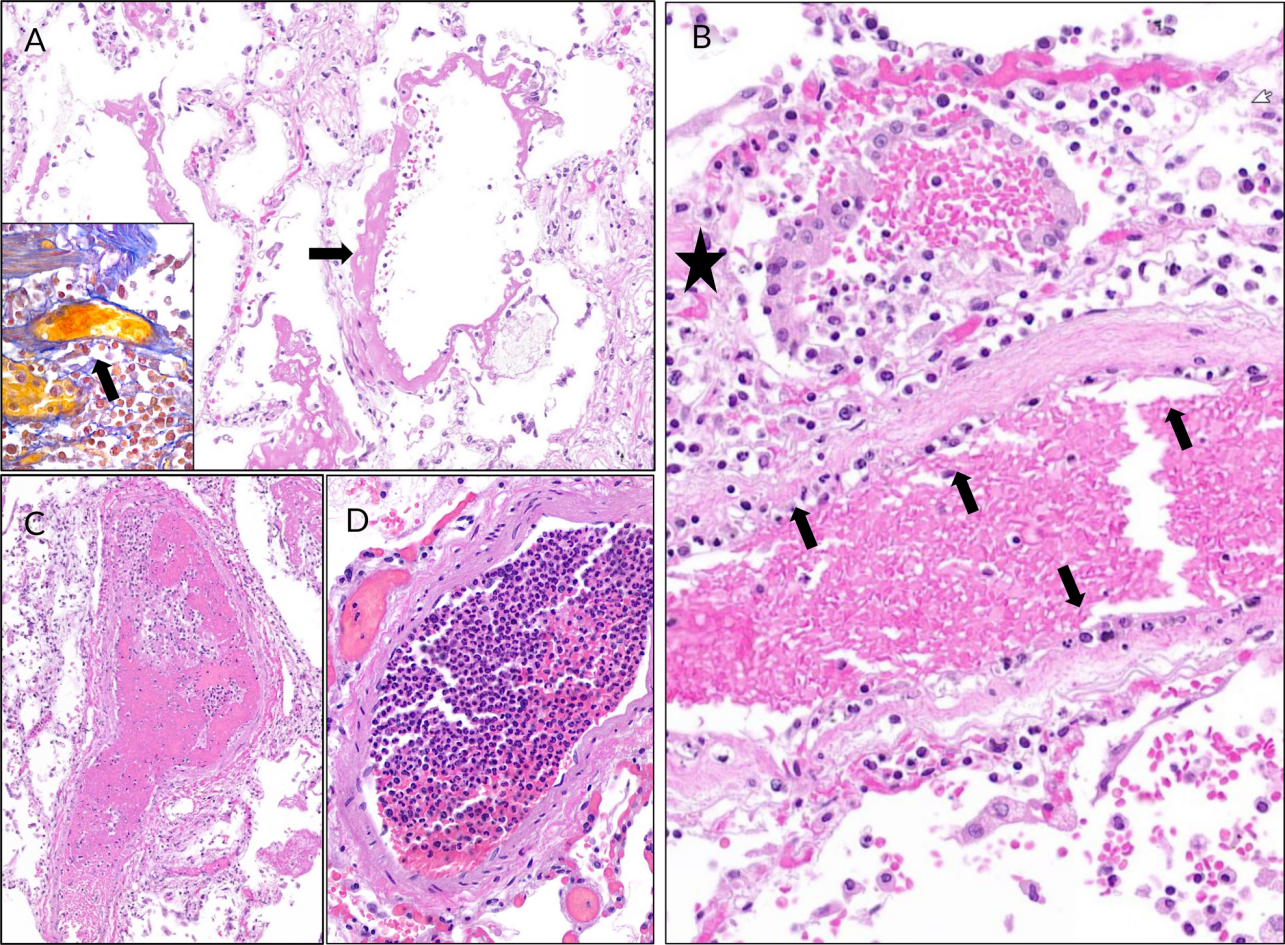
Histopathological findings in lungs of COVID‐19 patients. (A) Lung of a male COVID‐19 patient with pre‐existing lung fibrosis. Diffuse alveolar damage with hyaline membranes (arrow) and congested interstitial capillaries (H&E stain) are present. Inset: intra‐vascular fibrin formation in a small sized vessel (arrow) (SFOG trichrome stain). (B) Lung of a male COVID‐19 patient with hyperplastic type II alveolar epithelial cells (star), desquamation and pronounced endotheliitis of a medium sized pulmonary vessel (arrows). (C) Pulmonary fibrin thrombus in a male COVID‐19 patient in a medium sized arterial vessel (H&E stain). (D) Leucocytic intra‐vascular thrombus (‘leucocytic clot’) with minimal fibrin formation (H&E stain).

In contrast to our influenza autopsies, all COVID‐19 autopsy studies [7, 8, 9, 10, 11, 12, 13, 14, 15, 16] (Table 1) highlight the high incidence of thromboembolic events. Our previous autopsy study also documented cases with lung embolism and a characteristic COVID‐19 related endotheliitis in different organs [14] (Figures 3, 4, 5). Recently, Wichmann *et al* reported the findings of 12 consecutive, legally mandated autopsies of patients with COVID‐19 [7]. The authors noted a high incidence (42%) of pulmonary embolism with and without underlying deep venous thrombosis (DVT). Menter *et al* described four cases with prominent central pulmonary embolism in a series of 21 COVID‐19 autopsies [9]. Lax and colleagues performed autopsies on 11 patients randomly selected among 48 hospitalised COVID‐19 positive patients [8]. Visible pulmonary thrombi were noted in all cases with associated lung infarctions in 9 out of 11 autopsies (81%). Most of these patients had received prophylactic anticoagulant therapy, indicating that pulmonary thrombi were formed despite anticoagulant therapy. Clinical studies describe VTEs in 20–36% of COVID‐19 patients which is significantly higher than the prevalence of VTEs in influenza A patients [3, 17].

## Discussion

Both autopsy [7, 8, 9, 10, 11, 12, 13, 14, 15, 16] and clinical studies [3, 17] report a high incidence of thromboembolic events in COVID‐19 patients. A surprising aspect of our study on influenza‐associated fatalities from 1918 to 1919 was the complete absence of macroscopic thromboembolic events, e.g. grossly visible pulmonary thrombi, infarction of the kidney, lung, heart or intestine. Due to this unexpected finding, we re‐evaluated other influenza autopsy studies, including autopsies that were performed in the United States by prominent pathologists (e.g. Prof. Goodpasture) during the 1918 influenza pandemic (see review by Taubenberger *et al* [18]). Of note, macroscopic thromboembolic events, i.e. grossly visible pulmonary thrombi are also extremely rare. A very detailed autopsy series of 68 soldiers in U.S. army training camps dying before and during the 1918 influenza pandemic peak [19] revealed a complete absence of macroscopic thromboembolic events following gross examination. Pulmonary histological findings identified microscopic thrombus formation in only 5 of 68 patients (7%). Comparable to the Zurich series of 411 autopsies, all 68 soldiers had histological evidence of bacterial pneumonia, suggesting that the vast majority of influenza deaths resulted from secondary bacterial pneumonia. It has been speculated that these pulmonary deaths from pandemic influenza viruses resulted from poorly understood interactions between the infecting virus and secondary infections due to bacteria that colonise the upper respiratory tract. Coinfection with the influenza virus and bacteria may affect pathogenicity [20, 21].

Histopathological studies of the ‘Spanish’ influenza pandemic have already characterised the findings of diffuse alveolar damage (DAD). DAD describes the histological observations associated with adult respiratory distress syndrome (ARDS), which is due to the formation of hyaline membranes in alveoli, intra‐alveolar and interstitial oedema with inflammatory infiltrates, as well as capillary and small vessel thrombosis (Figure 1). Intra‐alveolar oedema and haemorrhage are outstanding features of influenza virus pneumonia. Necrosis of the alveolar wall was regarded as a consequence of capillary thrombosis. Most additional changes were related to secondary bacterial pneumonias.

Our study reports on complete influenza autopsies that occurred during the era prior to the use of antibiotics although, even in the era of antibiotics, later stages of influenza A virus pneumonia are almost always complicated by secondary pneumonia [18, 20]. In the modern era, the establishment of life‐prolonging intensive care unit treatment and anticoagulant prophylaxis make it more difficult than in 1918 to dissect the relationship between influenza A infection and thromboembolic events. The spectrum of histological findings in later pandemics or in deaths occurring during seasonal influenza outbreaks is similar to the histopathological changes described in the 1918/1919 influenza pandemic [18, 21]. The 1968 influenza pandemic was mild and autopsy studies were uncommon. In April 2009 the novel H1N1 influenza A virus (‘swine flu’) emerged and rapidly reached pandemic proportions. Autopsy studies of patients succumbing to the novel H1N1 influenza A virus were rare. Harms *et al* published an observational case study of pathological findings in eight patients and compared them with eight age‐, sex‐, body mass index (BMI)‐ and treatment‐matched control subjects [22]. DAD accompanied by acute bronchopneumonia was seen in six of these eight patients. Histology showed only peripheral pulmonary vascular thrombosis (related to DAD) in five of the eight patients with influenza and in three of the eight control subjects, but major thromboembolic findings were not reported in the autopsies [22, 23] .

In the era of standard mechanical ventilation, regular use of antibiotics as well as systematic prescription of thrombosis prophylaxis, the incidence of thromboembolic events and pulmonary thrombosis could be different. Van Wissen *et al* performed a large nested case–control study in patients with clinical suspicion of pulmonary embolism [24], but there was no association of influenza A infection with increased risk of acute pulmonary embolism.

Similar to COVID‐19 caused by SARS‐Cov‐2, severe acute respiratory syndrome (SARS), caused by SARS‐Cov‐1, is also associated with thrombotic complications [25]. This is in contrast to influenza. SARS occurred in Singapore in March 2003 [26, 27, 28]. A small autopsy series in Singapore revealed macroscopic pulmonary thrombemboli on gross examination in four of the eight SARS patients [29]. In addition, in five patients, there was evidence of peripheral microscopic thrombus formation by histology. This finding was confirmed in three patients from China [25, 30] who died from SARS. Systemic vasculitis with localised fibrinoid necrosis and infiltration of monocytes, lymphocytes and plasma cells into vessel walls was described in the heart, lung, liver, kidney, adrenal gland and muscles. Thrombosis was present in small veins. Post‐mortem tissue sample analyses from six other patients who died from SARS in 2003 did not report thrombosis or thromboembolic complications [31]. Multiorgan failure and DAD were suggested as main causes of death in SARS [21].

The presence of pulmonary microvascular thrombi was also noted histologically in most COVID‐19 autopsies [7, 9]. Our overview shows that microvascular thrombi are a characteristic feature of both COVID‐19 and influenza A, because DAD is the major finding in fatal cases with hypoxic respiratory failure (Figure 1). In addition, microthrombi are not specific to viral pneumoniae but rather a general feature of acute lung injury which can be also seen in bacterial pneumoniae, sepsis, trauma, aspiration and toxic inhalation. Clinically, a mild association of increased venous thromboembolism risk is reported in all patients with infections [32], as well as in patients with influenza A and ARDS [33].

There could be other factors that account for the differences we observed between patients with COVID‐19 and those with influenza. The patients in our autopsy series from 1918/1919 were young, and most of them where of normal weight or were underweight. Most COVID‐19 autopsies were from older patients suffering from obesity, type 2 diabetes mellitus or coronary heart disease (Table 1). Differences could also result from new treatments such as other standard mechanical ventilation procedures including pressure‐controlled ventilation and the length of hospitalisation since patients with influenza A remain stable at a significantly lower level more often than those with COVID‐19. The current systematic prescription of thrombosis prophylaxis may also impact these differences.

Apart from these differences, our direct comparison of macroscopic autopsy findings suggests a significantly greater degree of grossly visible pulmonary macrothrombi as well as lung infarctions in patients with COVID‐19 compared to influenza A autopsies even though most patients received empiric thromboprophylaxis. Moreover, Wichmann *et al* describe a high incidence of thrombi in prostatic venous plexus [7] and Lax *et al* report thrombosis within the pulmonary arterial circulation in the absence of DVT or VTEs [8]. This is consistent with a SARS‐related *de novo* coagulopathy with generalised *in situ* clot formation, which could explain the high incidence of pulmonary embolism with or without underlying DVT and in the absence of a history of VTEs.

Macrothrombus formation in lung arteries without a history of VTEs is unusual, but could be explained by the recent findings of Ackerman *et al* [5]. They compared seven lungs from patients who died from COVID‐19 with lungs from patients who died from ARDS secondary to influenza A (H1N1) infection. They measured significantly more capillary microthrombi and describe a specific microangiopathy (intussusceptive angiogenesis) in the lungs of COVID‐19 patients compared to influenza H1N1. This finding was explained by severe endothelial injury associated with intra‐cellular SARS‐CoV‐2 infection and a greater number of ACE2‐positive cells in the lungs of patients with COVID‐19. ACE2 is the host‐cell receptor for COVID‐19. Our comparison of influenza A and COVID‐19 autopsy findings supports the idea that there is a specific pathogenesis of severe COVID‐19 disease.

Endothelial cells play a central role in the vascular phase of COVID‐19 [14, 34, 35]. Pre‐existing endothelial dysfunction explains how patients with old age, obesity, hypertension and diabetes mellitus are at a higher risk for a fatal outcome when suffering from COVID‐19. The presence of SARS‐CoV‐2 within endothelial cells is a consistent finding suggesting that direct viral effects as well as systemic endotheliitis may contribute to formation of microthrombi in COVID‐19 [14, 35].

## Conclusions

The combination of systemic inflammation, endotheliitis in multiple organs, and *in situ* intra‐vascular coagulation may promote the formation of large‐vessel thrombosis leading to a high prevalence of thromboembolic events; finally resulting in multisystem organ failure in fatal COVID‐19 cases. A specific COVID‐19 related coagulopathy could explain the higher mortality rate of COVID‐19 in comparison to seasonal flu. However, further autopsy studies are important to identify the contributions of embolism and pulmonary thrombosis to the deaths of patients with SARS‐CoV‐2 infection. Importantly, the high number of arterial and VTE in COVID‐19 autopsies confirms the clinical need for DVT diagnostic strategies and thromboprophylaxis in COVID‐19 patients [3].

## Author contributions statement

NMBK, MH, UM, TH, ZV and HM were involved in data collection. NMBK, MH, TH, ZV and HM analysed and interpreted data and carried out the literature search. MH, ZV and HM generated figures. HM was responsible for the study design. All authors were involved in critical reading, writing the paper and had final approval of the submitted and published versions.

## References

[1] Zhu N , Zhang D , Wang W , *et al* A novel coronavirus from patients with pneumonia in China, 2019. N Engl J Med 2020; 382: 727–733. 31978945 10.1056/NEJMoa2001017 PMC7092803

[2] Chen N , Zhou M , Dong X , *et al* Epidemiological and clinical characteristics of 99 cases of 2019 novel coronavirus pneumonia in Wuhan, China: a descriptive study. Lancet 2020; 395: 507–513. 32007143 10.1016/S0140-6736(20)30211-7 PMC7135076

[3] Lodigiani C , Iapichino G , Carenzo L , *et al* Venous and arterial thromboembolic complications in COVID‐19 patients admitted to an academic hospital in Milan, Italy. Thromb Res 2020; 191: 9–14. 32353746 10.1016/j.thromres.2020.04.024 PMC7177070

[4] Petersen E , Koopmans M , Go U , *et al* Comparing SARS‐CoV‐2 with SARS‐CoV and influenza pandemics. Lancet Infect Dis 2020; 20: e238–e244. 32628905 10.1016/S1473-3099(20)30484-9 PMC7333991

[5] Ackermann M , Verleden SE , Kuehnel M , *et al* Pulmonary vascular endothelialitis, thrombosis, and angiogenesis in Covid‐19. N Engl J Med 2020; 383: 120–128. 32437596 10.1056/NEJMoa2015432 PMC7412750

[6] Koren NM . Die Spanische Grippe in Zürich 1918/19: Erfahrungen aus heutiger Sicht anhand von 970 Sektionen des Pathologischen Institutes Zürich. Department of Pathology and Molecular Pathology. Medical Faculty, University Zurich: Zurich, 2003 004604059.

[7] Wichmann D , Sperhake JP , Lutgehetmann M , *et al* Autopsy findings and venous thromboembolism in patients with COVID‐19. Ann Intern Med 2020; 173: 268–277. 32374815 10.7326/M20-2003 PMC7240772

[8] Lax SF , Skok K , Zechner P , *et al* Pulmonary arterial thrombosis in COVID‐19 with fatal outcome: results from a prospective, single‐center, clinicopathologic case series. Ann Intern Med 2020; 173: 350–361. 32422076 10.7326/M20-2566 PMC7249507

[9] Menter T , Haslbauer JD , Nienhold R , *et al* Post‐mortem examination of COVID19 patients reveals diffuse alveolar damage with severe capillary congestion and variegated findings of lungs and other organs suggesting vascular dysfunction. Histopathology 2020; 77: 198–209. 32364264 10.1111/his.14134 PMC7496150

[10] Buja LM , Wolf DA , Zhao B , *et al* The emerging spectrum of cardiopulmonary pathology of the Coronavirus disease 2019 (COVID‐19): report of 3 autopsies from Houston, Texas, and review of autopsy findings from other United States cities. Cardiovasc Pathol 2020; 48: 107233. 32434133 10.1016/j.carpath.2020.107233 PMC7204762

[11] Fox SE , Akmatbekov A , Harbert JL , *et al* Pulmonary and cardiac pathology in African American patients with COVID‐19: an autopsy series from New Orleans. Lancet Respir Med 2020; 8: 681–686. 32473124 10.1016/S2213-2600(20)30243-5 PMC7255143

[12] Barton LM , Duval EJ , Stroberg E , *et al* COVID‐19 Autopsies, Oklahoma, USA. Am J Clin Pathol 2020; 153: 725–733. 32275742 10.1093/ajcp/aqaa062 PMC7184436

[13] Sekulic M , Harper H , Nezami BG , *et al* Molecular detection of SARS‐CoV‐2 infection in FFPE samples and histopathologic findings in fatal SARS‐CoV‐2 cases. Am J Clin Pathol 2020; 154: 190–200. 32451533 10.1093/ajcp/aqaa091 PMC7314275

[14] Varga Z , Flammer AJ , Steiger P , *et al* Endothelial cell infection and endotheliitis in COVID‐19. Lancet 2020; 395: 1417–1418. 32325026 10.1016/S0140-6736(20)30937-5 PMC7172722

[15] Grimes Z , Bryce C , Sordillo EM , *et al* Fatal pulmonary thromboembolism in SARS‐CoV‐2‐infection. Cardiovasc Pathol 2020; 48: 1054–8807. 10.1016/j.carpath.2020.107227 PMC7214296 32718733

[16] Bosmuller H , Traxler S , Bitzer M , *et al* The evolution of pulmonary pathology in fatal COVID‐19 disease: an autopsy study with clinical correlation. Virchows Arch 2020; 477: 349–357. 32607684 10.1007/s00428-020-02881-x PMC7324489

[17] Poissy J , Goutay J , Caplan M , *et al* Pulmonary embolism in COVID‐19 patients: awareness of an increased prevalence. Circulation 2020; 142: 184–186. 32330083 10.1161/CIRCULATIONAHA.120.047430

[18] Taubenberger JK , Morens DM . The pathology of influenza virus infections. Annu Rev Pathol 2008; 3: 499–522. 18039138 10.1146/annurev.pathmechdis.3.121806.154316 PMC2504709

[19] Sheng ZM , Chertow DS , Ambroggio X , *et al* Autopsy series of 68 cases dying before and during the 1918 influenza pandemic peak. Proc Natl Acad Sci U S A 2011; 108: 16416–16421. 21930918 10.1073/pnas.1111179108 PMC3182717

[20] Morens DM , Taubenberger JK , Fauci AS . Predominant role of bacterial pneumonia as a cause of death in pandemic influenza: implications for pandemic influenza preparedness. J Infect Dis 2008; 198: 962–970. 18710327 10.1086/591708 PMC2599911

[21] Nickol ME , Kindrachuk J . A year of terror and a century of reflection: perspectives on the great influenza pandemic of 1918‐1919. BMC Infect Dis 2019; 19: 117. 30727970 10.1186/s12879-019-3750-8 PMC6364422

[22] Harms PW , Schmidt LA , Smith LB , *et al* Autopsy findings in eight patients with fatal H1N1 influenza. Am J Clin Pathol 2010; 134: 27–35. 20551263 10.1309/AJCP35KOZSAVNQZW

[23] Shelke VN , Kolhapure RM , Kadam D , *et al* Pathologic study of pandemic influenza A (H1N1) 2009 cases from India. Pathol Int 2012; 62: 36–42. 22192802 10.1111/j.1440-1827.2011.02751.x

[24] van Wissen M , Keller TT , Ronkes B , *et al* Influenza infection and risk of acute pulmonary embolism. Thromb J 2007; 5: 16. 17939867 10.1186/1477-9560-5-16 PMC2104525

[25] Nicholls JM , Poon LL , Lee KC , *et al* Lung pathology of fatal severe acute respiratory syndrome. Lancet 2003; 361: 1773–1778. 12781536 10.1016/S0140-6736(03)13413-7 PMC7112492

[26] Rota PA , Oberste MS , Monroe SS , *et al* Characterization of a novel coronavirus associated with severe acute respiratory syndrome. Science 2003; 300: 1394–1399. 12730500 10.1126/science.1085952

[27] Drosten C , Gunther S , Preiser W , *et al* Identification of a novel coronavirus in patients with severe acute respiratory syndrome. N Engl J Med 2003; 348: 1967–1976. 12690091 10.1056/NEJMoa030747

[28] Ksiazek TG , Erdman D , Goldsmith CS , *et al* A novel coronavirus associated with severe acute respiratory syndrome. N Engl J Med 2003; 348: 1953–1966. 12690092 10.1056/NEJMoa030781

[29] Chong PY , Chui P , Ling AE , *et al* Analysis of deaths during the severe acute respiratory syndrome (SARS) epidemic in Singapore: challenges in determining a SARS diagnosis. Arch Pathol Lab Med 2004; 128: 195–204. 14736283 10.5858/2004-128-195-AODDTS

[30] Ding Y , Wang H , Shen H , *et al* The clinical pathology of severe acute respiratory syndrome (SARS): a report from China. J Pathol 2003; 200: 282–289. 12845623 10.1002/path.1440 PMC7168017

[31] Gu J , Gong E , Zhang B , *et al* Multiple organ infection and the pathogenesis of SARS. J Exp Med 2005; 202: 415–424. 16043521 10.1084/jem.20050828 PMC2213088

[32] Schmidt M , Horvath‐Puho E , Thomsen RW , *et al* Acute infections and venous thromboembolism. J Intern Med 2012; 271: 608–618. 22026462 10.1111/j.1365-2796.2011.02473.x PMC3505369

[33] Obi AT , Tignanelli CJ , Jacobs BN , *et al* Empirical systemic anticoagulation is associated with decreased venous thromboembolism in critically ill influenza A H1N1 acute respiratory distress syndrome patients. J Vasc Surg Venous Lymphat Disord 2019; 7: 317–324. 30477976 10.1016/j.jvsv.2018.08.010

[34] Teuwen LA , Geldhof V , Pasut A , *et al* COVID‐19: the vasculature unleashed. Nat Rev Immunol 2020; 20: 389–391. 32439870 10.1038/s41577-020-0343-0 PMC7240244

[35] Puelles VG , Lutgehetmann M , Lindenmeyer MT , *et al* Multiorgan and renal tropism of SARS‐CoV‐2. N Engl J Med 2020; 383: 590–592. 32402155 10.1056/NEJMc2011400 PMC7240771

